# The Effect of Nordic Walking Intervention (NORDIN-JOY) on Individuals with Intellectual Disabilities and Their Families: A Multicenter Randomized Crossover Study

**DOI:** 10.3390/clinpract15030053

**Published:** 2025-03-06

**Authors:** Daniel González-Devesa, Carlos Ayán-Pérez, Eva González-Devesa, Jose Carlos Diz-Gómez

**Affiliations:** 1 Grupo de Investigación en Actividad Física, Educación, y Salud (GIAFES), Universidad Católica de Ávila, C/Canteros, 05005 Ávila, Spain; 2 Well-Move Research Group, Galicia Sur Health Research Institute (IIS Galicia Sur), SERGAS-UVIGO, 36310 Vigo, Spain; cayan@uvigo.es (C.A.-P.); jcdiz@uvigo.gal (J.C.D.-G.); 3 Departamento de Didácticas Especiáis, Universidade de Vigo, 36310 Vigo, Spain; 4 Servicio de Nutrición, Centro de Salud de Cee, 15270 A Coruña, Spain; eva.gonzalez.devesa@sergas.es

**Keywords:** physical fitness, health promotion, rehabilitation, caregivers, body composition, adherence

## Abstract

Background: We aim to evaluate the effect of the Nordic Walking program on the quality of life and functionality of individuals with intellectual disabilities; Methods: The NORDIN-JOY study is designed as a multicenter, randomized crossover trial. Participants in the experimental group will engage in a Nordic Walking intervention, while those in the control group will participate in a Fit 5-Fitness Cards intervention. Both physical training programs will consist of two 60 min sessions per week over a three-month period. The study will assess measures of quality of life and physical fitness. Additionally, the feasibility and cost-effectiveness of both programs, as well as the average weekly physical activity levels during the six months following the intervention, will be evaluated; Results: The results of this study are anticipated to provide valuable insights into the effects of structured exercise interventions on individuals with intellectual disabilities. These findings will be disseminated through peer-reviewed journals and academic conferences; Conclusions: This protocol seeks to establish evidence on the impact of exercise programs in individuals with intellectual disabilities. The findings have the potential to inform specific recommendations for healthcare professionals, caregivers, and policymakers, promoting physical activity as a cost-effective strategy for improving care and quality of life in this population.

## 1. Introduction

According to data from the 2019 Global Burden of Disease study, an estimated 107.62 million individuals worldwide (1.74% of the population) have an intellectual disability (ID) [1].

Multiple factors shape the strategic planning of care for this population. Notably, the rising life expectancy, projected to reach 69.7 years in these nations [2], necessitates the provision of sustained and appropriate support systems throughout the lifespan, particularly for adults with ID who may outlive their primary caregivers [3]. On the other hand, premature aging, typically beginning around the age of 45, is associated with an increased prevalence of chronic conditions, such as chronic constipation, thyroid disorders, and obesity, often linked to sedentary behavior [4]. Notably, adults with ID experience functional decline as early as the fourth decade of their life, as a consequence of premature aging, as well as early signs of cognitive decline [5].

According to age-standardized mortality ratio data, 31.7% of recorded deaths among individuals with IDs were deemed avoidable, with 21.1% classified as treatable and 19.9% as preventable. These findings highlight the urgent need for targeted preventive strategies within this population [6]. In this regard, physical activity emerges as a key strategy for enhancing quality of life (QoL) and health, promoting active and healthy aging [7]. Engaging in physical activity provides numerous benefits for individuals with ID, including improved cardiorespiratory fitness, increased muscular strength, healthy weight management, and a reduced risk of high blood pressure, excessive weight gain, stroke, and elevated blood sugar levels [8].

In addition to this, it should be noted that physical fitness (PF) has been found to be predictive of a decline in daily function in the general population and in older adults with ID [9]. In accordance with this, it seems important to develop strategies aimed at improving health-related PF, which is a well-known marker of health that is considered to be an important predictor of morbidity and mortality for cardiovascular disease and for all causes [10]. Traditionally, body composition, cardiorespiratory and muscular fitness and flexibility, are considered the main components of health-related PF [11]. In this context, it is essential for both public and private authorities to develop prevention strategies centered on the promotion of physical activity, which is key to improving PF in individuals with ID. Such strategies should be designed to overcome common exercise barriers reported by individuals with ID, including economic cost, transportation issues, and lack of facilities [12]. Additionally, since exercise barriers may vary depending on the severity of ID, it is essential to promote activities that are motivating, enjoyable, and easy to perform [13].

Nordic Walking (NW) is proposed as an effective therapeutic approach, as walking is the most common form of exercise within this population [14]. The ability to walk is closely linked to daily physical fitness, cardiovascular health, and long-term independence. NW could serve as a valuable therapeutic intervention for this population for several reasons. Firstly, NW is regarded as a simple and feasible form of physical activity that can be performed by almost anyone, at any time, and in nearly any location [15]. Secondly, NW has been shown to have beneficial physical and psychological effects, including improvements in QoL [16]. Lastly, NW can be performed alongside family members and friends, enabling group exercise, which is likely to enhance adherence to this form of physical activity [17].

To enhance exercise adherence, it is essential to identify activities that provide a clear and motivating structure. In this sense, the Fit 5-Fitness Cards program seems to be an interesting option. This program was created with the aim of promoting regular physical activity, nutrition and hydration among Special Olympic athletes. The Fitness Cards program provides a comprehensive set of exercises targeting four key fitness components: endurance, strength, flexibility, and balance. These exercises are structured across multiple levels, allowing individuals to progress at their own pace and adapt routines to their specific abilities [18]. This approach facilitates the involvement of both participants and their caregivers, fostering autonomy and promoting a healthier lifestyle. Previous research has demonstrated that the Fit 5-Fitness program is an effective and appealing intervention, yielding significant improvements in various health metrics, including reductions in blood pressure and resting heart rate [19]. Furthermore, both the Fit 5-Fitness Cards program and NW could positively contribute to reducing obesity in individuals with ID [19,20]. These findings are particularly critical given the high prevalence of obesity in individuals with ID and the pressing need for multifactorial strategies that encourage physical activity to mitigate this issue.

Despite the interest in physical activity approaches, there remains a gap in research regarding their effectiveness in enhancing functionality and QoL among individuals with ID. While scientific literature has confirmed the benefits of NW in various populations, such as healthy older adults [21], and individuals with metabolic pathologies [22], studies on its potential impact on ID are scarce. To the best of the authors’ knowledge, existing research has primarily focused on body composition, balance and gait parameters in individuals with Down syndrome [23,24]. The only known study examining the effects of NW in this population assessed its impact on body composition and physical fitness in 12 obese women with ID [25]. Therefore, further research is needed to explore the broader benefits of NW for individuals with ID.

Regarding the Fit 5-Fitness Cards, the limited existing research has demonstrated their effectiveness in improving body composition and metabolic health among Special Olympic athletes [19]. However, only one study appears to have been conducted specifically with individuals with ID. In the study by Ptomey et al. [26], the Fit 5-Fitness Cards were utilized to promote physical activity among adults with ID as part of a comprehensive weight management intervention. This highlights the clear need for further research to evaluate the efficacy of this strategy in individuals with ID.

Therefore, the primary objective of this study is to evaluate the effect of the NW program on the QoL and functionality of individuals with ID. Additionally, the secondary objectives include assessing the impact of the Fit 5-Fitness Cards program on QoL and functionality, as well as comparing the safety, feasibility, and adherence of these two alternative exercise interventions.

## 2. Materials and Methods

### 2.1. Study Design

**Figure 1:**
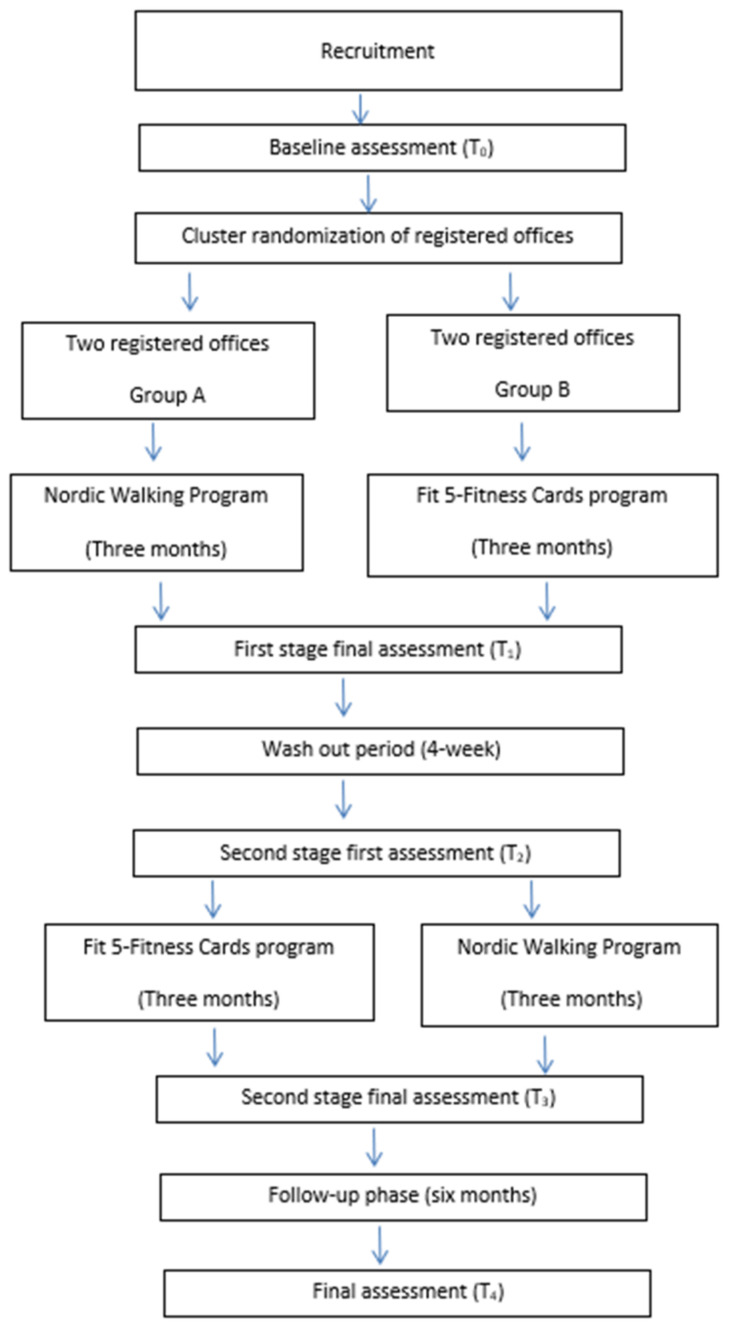
Flow chart of the study.

This is a pilot study employing a crossover, cluster-randomized design with repeated measures. This design allows for a within-subject comparison, reducing inter-individual variability and increasing statistical efficiency compared to parallel-group trials, where each subject receives only one treatment [27]. Participating centers will be randomized in a 1:1 ratio into two groups. Two sequential interventions (NW program and Fit 5-Fitness Cards) will be conducted, separated by a 4-week washout period to mitigate potential carryover effects [27]. Following the washout period, the interventions will be repeated in reverse order, adhering strictly to the established protocol. A comprehensive outline of the design and procedures is presented in Figure 1.

### 2.2. Participants

The sample size was estimated using G*Power (version 3.1., University of Düsseldorf, City, Germany) [28]. A priori power analysis, based on the Wilcoxon signed-rank test, was conducted with an alpha level of 0.05, statistical power of 0.80, and a moderate effect size (Cohen’s d = 0.5). The analysis determined that 67 participants were required. To account for potential dropouts, the final sample size was set at 100 participants, with recruitment evenly distributed across four centers (approximately 25 participants per center). This design ensures robust data collection while allowing for comparative analysis of the interventions.

Participants for this study will be recruited through invitation letters distributed via four associations located across Spain. Eligibility criteria for inclusion in the study are as follows: (a) a confirmed diagnosis of ID arising from neurodevelopmental disorders or autism spectrum disorders; (b) classification of moderate or mild ID (based on a combination of IQ (Intellectual Quotient) scores and adaptive behavior), as defined by the criteria of the Spanish National Health System; and (c) Independent ambulatory ability.

The legal representatives or caregivers of potential participants will be responsible for consulting with the individuals, regarding their willingness to participate in the study and providing written consent on their behalf. Additionally, all participants will have the opportunity to express their own consent or assent regarding their involvement [29].

### 2.3. Procedures

The registered sites will be randomly assigned to two groups using a random number table generated in Excel. The randomization process will be unrestricted, with no stratification or pairing of sites. To minimize selection bias, allocation concealment will be ensured by having an independent researcher generate the randomization sequence and store it in a secure file. Following the recruitment of the participating sites and the completion of the training period for monitors/caregivers, the randomization table will be unblinded, and the sites will be allocated to one of two groups. Preferably, the monitors/caregivers will be occupational therapists, physiotherapists, or social workers with specialized training in adapted exercise.

Group A will initially implement the NW program, followed by the Fit 5-Fitness Cards program. Conversely, Group B will undertake the programs in the reverse order. The random sequence will be generated by J.C.D-G., while D.G-D. will be responsible for informing the registered sites of the exercise sequence their members are required to follow.

### 2.4. Measurements

To assess the effects of the training programs, functionality tests will be selected based on the following criteria: (a) proven feasibility for individuals with ID; (b) ease of execution with limited resources; and (c) the ability to perform the tests within the registered facilities.

Participants will receive a detailed explanation of the protocols, be allowed to practice the tests in advance, and receive guidance throughout the assessment process. Neither the monitors nor the participants will be blinded to the interventions.

#### 2.4.1. Quality of Life

The KidsLife Scale, a questionnaire specifically designed for Spanish individuals with ID, will be used to assess the impact of both programs on participants’ QoL. This scale consists of 96 items organized into eight QoL domains and must be completed by a family member or caregiver. When administered to a sample of young 121 individuals with ID, the scale demonstrated good internal consistency, with Cronbach’s alpha values ranging from 0.812 to 0.949. Additionally, construct validity evidence provided by Confirmatory Factor Analysis indicated adequate fit indices [30].

#### 2.4.2. Functionality

- Dynamic Balance and Gait Speed: The “Timed Up and Go Test” (TUGT) will be employed, a reliable assessment tool for individuals with ID [31]. In a sample of 31 adults with ID, the TUG proved to be a feasible assessment tool, demonstrating excellent test–retest reliability (ICC: 0.90; 95% CI: 0.82–0.95) [32];
- Flexibility: The sit and reach test (SR) will be used to assess the impact of the program on the participants’ lower-body flexibility. For performing the SR, the participants will sit on the floor without shoes and with knees straight, and feet placed flat against the front-end panel of a standard SR box. Then, they will be asked to slowly reach forward as far as possible while placing the palms down along the measuring scale placed on the top of the box and to hold the position for approximately 2 s. The most distant point reached with the fingertips will be recorded (to the nearest centimeter). The best of two trials will be retained for analysis. The SR has demonstrated high reliability in a sample of 63 adolescents with ID (ICC = 0.97) [33] and has been previously used to assess fitness levels in adults with ID [34];
- Cardiorespiratory Fitness: Heart rate (HR) data will be collected using a Polar HR monitor (Polar Team Pro for iPad, version 1.0.1) during the tests. This method has been previously applied to individuals with ID [3].

#### 2.4.3. Feasibility of the Programs

The following variables would be recorded by the people who will administer the exercise programs to assess their feasibility: recruitment rate (number of participants recruited from those that fulfilled the inclusion criteria) [35]; attrition (number of drop-outs); completion rate (number of participants who completed each outcome measure); adherence measurement with comprehensive reporting [36] (proportion of participants with participation rates exceeding 80%); participation (total of exercise hours completed divided by the total number of possible hours); and safety and tolerability (number of participants who experienced exercise-related adverse effects, such as pain, dizziness, intense fatigue, falls, etc., resulting from exercise performance). Participants may experience some discomfort, fatigue, or muscle soreness, which are common among sedentary individuals starting a physical activity intervention. However, these effects typically diminish after two or three sessions [37].

#### 2.4.4. Cost-Effectiveness of the Program

To assess the cost-effectiveness of the intervention [38], we will use a cost-effectiveness ratio (CER), which quantifies the relationship between the total cost of the intervention and the improvement in the primary outcomes. In addition, the CER of improvement in QoL and functionality will be determined using the following formula:

$$CER=\frac{TC}{\Delta E}$$

where

- TC = Total cost of the program (NW or Fit 5-Fitness Cards);
- ΔE = Change in average outcomes scores (post-intervention minus pre-intervention).

The incremental cost-effectiveness ratio (ICER) will be computed to compare the economic and functional impact of NW against the Fit 5-Fitness Cards program. The ICER will be determined using the following equation [39]:

$$ICER=\frac{C1-C0}{E1-E0}$$

where

- C1 = Total cost of the NW intervention;
- C0 = Total cost of the Fit 5-Fitness Cards program;
- E1 = Post-intervention outcome for the NW group (e.g., average QoL score);
- E0 = Post-intervention outcome for the Fit 5-Fitness Cards group.

#### 2.4.5. Post-Intervention Average Weekly Physical Activity

Before the intervention begins, the participants and their parents will be asked to write the main physical activities (modality and duration) that they usually perform together in a typical week. Once the intervention ends (7 months after the start of the exercise program), a physical activity log will be given to the participants and their parents. They will be asked to register any type of physical activity (modality and duration) performed during a typical week of each month for the following six months.

Four NW poles will be given to the participants (two) and their parents (two) who complete the whole intervention. They will be encouraged to continue practicing NW by themselves for the next six months. Additionally, the registered offices that took part in the research will allow the use of the facilities in which the Fit 5-Fitness Cards and NW program took place at least two days per week, so that the participants and their parents could keep performing Fit 5-Fitness Cards and NW activities, also for the next six months.

Health-related PF, feasibility and QoL measurements will be carried out one week before the start of the training programs (To), once a week after the completion of the first stage (T1), one month later (T2), at the end of the second phase (T3), and six months later (T4). Record logs will be returned at T0 and T4. All the field-based tests and the questionnaire will be administered by the people in charge of the exercise intervention performed in each ID registered office.

### 2.5. Intervention

Before the intervention allocation is revealed, the monitors/caregivers will receive training on both interventions to ensure that the explanations and implementation are as consistent as possible across all centers.

Both exercise training programs would be performed at the rate of two 60 min sessions per week for three months. After the wash-out period (one month), the training programs will be performed in reverse order for another three months. A follow-up phase of six months is scheduled once the intervention has finished (7 months).

#### 2.5.1. Nordic Walking

Participants will take part in an NW program performed two times a week in 60 min sessions, that will be carried out on non-continuous days, for three months, following previous procedures [15]. Given that any walking technique with poles elicits higher metabolic responses and muscular activation than walking [40], the first four sessions of the program will be focused on learning the NW technique (four sessions of 60 min). Each session will comprise a warm-up of 10 min, a core period (40 min of NW) and a cool-down period of 10 min. The progressive increase in workload will be conducted by an increase in distance and velocity scope (from 2.500 m and 3.75 km/h to 3.500 km and 5.25 km/h) without modifying the core period duration (40 min). In all cases, the intensity of activity will remain less than 75% of the maximal heart rate, a value that will be assessed by means of the equation developed by Fernhall et al. [41]. The NW pole length will be adjusted to the standard formula (0.65 × height) to ensure adequate technique performance.

#### 2.5.2. Fit 5-Fitness Cards

The Fit 5-Fitness Cards program is a structured approach focusing on four key areas of physical fitness: endurance, strength, flexibility, and balance. The sessions include simple exercises tailored to different skill levels, organized into progressive stages (levels 1 to 5) to match the abilities of each participant [18]. Participants will engage in 60 min sessions conducted on non-consecutive days over the course of three months.

The sessions will be led by a graduate in Sport Science with over six years of experience training special populations. Additionally, an instructional video will be provided to support users in performing the exercises during each session. This will ensure consistency across the different centers.

### 2.6. Statistical Analysis

Data will be reported as medians and interquartile ranges for non-normally distributed variables (checked with the Kolmogorov-Smirnoff test) or as means and standard deviations for normally distributed variables. Qualitative variables will be reported as n (%). The intra-cluster correlation coefficient and the cluster-specific effects will also be calculated. Inter-group quantitative variables will be compared, including the primary outcome, with an unpaired, two-tailed t test or the Mann–Whitney U test, as applicable. For inter-group dichotomous data comparison, the two-tailed chi-square tests will be used, with the Yates correction or Fisher’s exact test as appropriate. For repeated measures analysis, the two-way analysis of variance with the Bonferroni correction or the Friedman test, as appropriate, will be used. Comparison will be adjusted by cluster effect. Subgroup analysis of the primary outcome will include sex, age, BMI (normal, overweight and obese), level of physical activity (Inactive, Active), and level of ID (mild, moderate, severe). Multivariate regression models will adjust for confounders such as sex, age, BMI, physical activity, and ID level. Multicollinearity will be checked using the Variance Inflation Factor (VIF), with VIF > 5 indicating high collinearity [42]. Sensitivity analyses will assess robustness. Statistical significance will be set at a two-tailed *p*-value of less than 0.05. All analyses will be conducted using SPSS v24.0 software (IBM Corp., Armonk, NY, USA).

## 3. Results

We expect that the proposed exercise program will improve the health-related physical fitness level of the participants, as well as they will increase their level of social participation. These results, in turn, would lead to a greater QoL. Overall, this research is meant to allow participants to acquire an active lifestyle by integrating the performance of new physical exercise modalities into their everyday lives.

If this goal is met and the obtained results can be published in international journals, ID associations could have real information regarding the feasibility and effects of new/alternative exercise modalities that can be put into practice in their own settings. Thus, these associations will find a new path or strategy for promoting the performance of physical activity among people with ID by means of performing low-cost and easy-to-do exercise modalities.

## 4. Discussion

People with ID have been shown to have low levels of physical activity practice [43]. In this regard, the performance of physical exercise among this population is considered a useful strategy, due to the positive impact that it has on their health [7]. Therefore, the primary objective of this study is to evaluate the effect of the NW program on the QoL and functionality of individuals with ID. Additionally, the secondary objectives include assessing the impact of the Fit 5-Fitness Cards program on QoL and functionality, as well as comparing the safety, feasibility, and adherence of these two alternative exercise interventions.

The expected results of this research are that NW will improve fitness and QoL among adults with ID. These findings have important policy implications. Policymakers should integrate NW into health promotion initiatives, allocate funding for accessible programs, and incorporate it into disability services and rehabilitation efforts. Training for caregivers and professionals should be prioritized to ensure safe implementation, while urban planning should support accessible walking paths. Embedding NW into public health policies can promote inclusivity and improve the well-being of individuals with disabilities.

In this context, it should be acknowledged that implementing NW programs requires tailored approaches to accommodate different ability levels. Disability service providers and community organizations should offer structured sessions, with trained caregivers and professionals to ensure safety and engagement. Adaptive techniques, individualized pacing, and social participation should be emphasized to maximize benefits. By following these recommendations, practitioners can enhance the overall health and QoL of adults with ID.

Previous studies have demonstrated that exercise interventions can effectively improve the QoL [44] and physical fitness [44,45,46] of an individual with ID. However, significant uncertainty remains regarding the effects of NW and the Fit 5-Fitness Cards program. The available evidence has primarily focused on a narrow range of outcomes, which may hinder the effective application of these findings in clinical settings. Therefore, it is essential to provide a comprehensive synthesis and evaluation of the current evidence.

This is the first randomized crossover study designed to evaluate the effects of physical activity interventions on individuals with a confirmed diagnosis of ID stemming from neurodevelopmental disorders or autism spectrum disorders. The objective of this protocol is to generate robust and reliable evidence on the impact of structured exercise programs in this population. The findings have the potential to guide specific recommendations for healthcare professionals, caregivers, and policymakers, highlighting physical activity as a cost-effective strategy to enhance care and improve the QoL for individuals with ID. Nevertheless, certain limitations may arise. One potential challenge is ensuring adherence to the program, as participants may initially lack motivation to engage in exercise. To address this, we anticipate that both NW and the Fit 5-Fitness Cards will be enjoyable activities. Nevertheless, we acknowledge the possibility of a decline in adherence during the follow-up phase, as the NW program will be unsupervised. Another potential limitation concerns the assessment of QoL, as the questionnaire will be completed by caregivers or family members rather than the participants themselves. Furthermore, the subjective nature of this outcome could affect the strength of the findings. Lastly, it should be mentioned that physical activity levels will be monitored through self-reported data, a subjective method prone to recall bias, especially when assessing sedentary behavior [47].

The expected results will also set the basis for further research. In this regard, future studies should explore the long-term effects of NW on physical and mental health outcomes among adults with ID taking into account severity levels. In addition, longitudinal studies could assess whether sustained participation leads to lasting improvements in mobility, cardiovascular health, and psychological well-being. Finally, comparative studies could examine NW against other physical activities to determine its relative effectiveness in promoting fitness and QoL.

## Data Availability

The original contributions presented in this study are included in the article. Further inquiries can be directed to the corresponding author.
